# Retrospective and comparative analysis of ^99m^Tc-Sestamibi breast specific gamma imaging versus mammography, ultrasound, and magnetic resonance imaging for the detection of breast cancer in Chinese women

**DOI:** 10.1186/s12885-016-2537-1

**Published:** 2016-07-11

**Authors:** Xiuyan Yu, Guoming Hu, Zhigang Zhang, Fuming Qiu, Xuan Shao, Xiaochen Wang, Hongwei Zhan, Yiding Chen, Yongchuan Deng, Jian Huang

**Affiliations:** Department of Surgical Oncology, Second Affiliated Hospital, Zhejiang University School of Medicine, Zhejiang University, Hangzhou, 310009 China; Department of Gynecology, Second Affiliated Hospital, Zhejiang University School of Medicine, Hangzhou, 310009 China; Department of Medical Oncology, Second Affiliated Hospital, Zhejiang University School of Medicine, Hangzhou, 310009 China; Department of Nuclear Medicine, Second Affiliated Hospital, Zhejiang University School of Medicine, Hangzhou, 310009 China

**Keywords:** Breast specific gamma imaging, Breast cancer, Sensitivity, Specificity

## Abstract

**Background:**

Diagnosing breast cancer during the early stage may be helpful for decreasing cancer-related mortality. In Western developed countries, mammographies have been the gold standard for breast cancer detection. However, Chinese women usually have denser and smaller-sized breasts compared to Caucasian women, which decreases the diagnostic accuracy of mammography. However, breast specific gamma imaging, a type of molecular functional breast imaging, has been used for the accurate diagnosis of breast cancer and is not influenced by breast density. Our objective was to analyze the breast specific gamma imaging (BSGI) diagnostic value for Chinese women.

**Methods:**

During a 2-year period, 357 women were diagnosed and treated at our oncology department and received BSGI in addition to mammography (MMG), ultrasound (US) and magnetic resonance imaging (MRI) for diagnostic assessment. We investigated the sensitivity and specificity of each method of detection and compared the biological profiles of the four imaging methods.

**Results:**

A total of 357 women received a final surgical pathology diagnosis, with 168 malignant diseases (58.5 %) and 119 benign diseases (41.5 %). Of these, 166 underwent the four imaging tests preoperatively. The sensitivity of BSGI was 80.35 and 82.14 % by US, 75.6 % by MMG, and 94.06 % by MRI. Furthermore, the breast cancer diagnosis specificity of BSGI was high (83.19 % vs. 77.31 % vs. 66.39 % vs. 67.69 %, respectively). The BSGI diagnostic sensitivity for mammographic breast density in women was superior to mammography and more sensitive for non-luminal A subtypes (luminal A vs. non-luminal A, 68.63 % vs. 88.30 %).

**Conclusions:**

BSGI may help improve the ability to diagnose early stage breast cancer for Chinese women, particularly for ductal carcinoma in situ (DCIS), mammographic breast density and non-luminal A breast cancer.

## Background

Breast cancer is the leading type of new cancer cases and the second leading cause of cancer related deaths in females worldwide [1]. During the past 30 years, the incidence and mortality of breast cancer in Chinese women has gradually increased and has become a primary cause of death, with more than 1.6 million people diagnosed and 1.2 million people dying of the disease each year [2, 3]. The current guidelines suggest that breast cancer screening and diagnostic imaging modalities include mammography (MMG) and ultrasound (US) for women at average risk and magnetic resonance imaging (MRI) for high-risk women [4]. These methods can detect early stage breast cancer and reduce mortality. Despite their effectiveness, these traditional imaging methods have limitations that complicate the standardization of image quality and can affect the diagnostic accuracy of the breast examination. The diagnostic accuracy of MMG is affected by mammographic breast density, with decreased sensitivity in patients with dense breasts [5]. For MRI, a variable degree of background parenchyma enhancement (BPE) of normal fibro-glandular tissue occurs. Marked BPE can cause a higher abnormal interpretation rate and may influence the accuracy of MRI [5, 6]. Notably, the mean age at diagnosis of breast cancer in China is 45–55 years, which is considerably younger than for Western females. Young women usually have a smaller proportion of fat content relative to the fibro-glandular tissue in their breasts compared to older, Chinese women, who usually have denser and smaller-sized breasts compared to Caucasian women [2]. Therefore, the traditional imaging modalities have a low diagnostic value in China.

Breast specific gamma imaging (BSGI) is a physiologic approach to breast imaging using a high resolution, small-gamma camera and a tracer agent called ^99m^Tc-Sestamibi (MIBI), and molecular breast imaging has significantly improved in recent years with the development of breast optimized imaging [7, 8]. MIBI retention in tumor cells is determined by the cellular and mitochondrial membrane potential and the presence of an ATP-powered efflux pump, such as P-glycoprotein, which can transport foreign substances out of cells. However, unlike MMG, BSGI performance is independent of breast density [7, 9, 10]. Furthermore BPE is likely related to the blood volume and vascular permeability of normal breast tissue; therefore, it is predicted not to influence the background MIBI uptake. The sensitivity (Se) and specificity (Sp) of BSGI from a meta-analysis of 8 studies, including 2183 lesions, were 95 % (95 % CI 93–96 %) and 80 % (95 % CI 78–82 %), respectively, and were not affected by the breast density [11]. This was better than the reported sensitivity and specificity for the largest breast MRI study (*n* = 821), with 88.1 % (95 % CI, 84.6–91.1 %) and 67.7 % (95 % CI, 62.7–71.9 %), respectively [12]. Therefore, the Society of Nuclear Medicine (SNM) recommended BSGI particularly for breast patients with breasts technically too difficult to examine using conventional mammography, including radiodense breast tissue, implants, free silicone, or paraffin injections [13].

Chinese women have denser breasts, and recent research also indicated that women with higher breast density are at an increased risk of breast cancer, and this is one of the highest risk factors for the prediction of breast cancer risk. Therefore, a useful and accurate breast imaging method is necessary. The development of a dedicated breast gamma imaging system has overcome these limitations and has returned scintimammography to the forefront of breast imaging. This was a retrospective study analyzing BSGI performed as an adjunct imaging method for Chinese women to detect breast cancer.

## Methods

### Patients

The hospital ethics committee approved this study. Written informed consent was obtained from each patient. A total of 357 breast disease patients who were diagnosed and treated at the oncology department (Second Affiliated Hospital of Zhejiang University School of Medicine, Hangzhou, China) from June 2012 to January 2015 were included in this single-institution study. Patients were first identified by reviewing the BSGI database, and 357 patients who underwent BSGI were reviewed. The including criteria were as follows: 1) female patients 18-years-old and older; 2) pathological proof of non-metastatic breast cancer; and 3) received ultrasound (US), mammography (MMG) and breast-specific gamma imaging (BSGI) before diagnosis. Clinicopathological characteristics, including age, menstrual state, histological type, grade, hormone receptors, HER2 and the clinical stage at diagnosis were obtained from the medical files at our institution and were included in a unique dedicated database.

### Imaging and pathologic review

#### Pathologic review

The histological type and grade were defined using the World Health Organization classification system. ER and PR tumor status are normally determined by immunohistochemistry (IHC) testing. Samples that have at least 1 % of cells staining positive for ER are considered ER-positive. Breast cancer tumors are classified as HER2-positive if they are scored as a 3 or more by an IHC method defined as a uniform membrane staining for HER2 in 10 % or more of tumor cells or have demonstrated HER2 gene amplification by a fluorescence in situ hybridization (FISH) method (single probe, average HER2 copy number ≥ 6.0 signals/cell; dual probe HER2/CEP17 ratio ≥ 2.0 with an average HER2 copy number ≥ 4.0 signals/cell; dual probe HER2/CEP17 ratio ≥ 2.0 with an average HER2 copy number < 4.0 signals/cell; HER2/CEP17 ratio < 2.0 with an average HER2 copy number ≥ 6.0 signals/cell) [14].

#### Ultrasound review

For the ultrasound examination, we used high-end equipment (IU Elite®; Philips Healthcare, Best, Netherlands), and all findings were documented in two perpendicular planes. The ultrasound positives were defined by an expert as highly suspected and suggestive of biopsy or operation.

#### Mammography review

The mammographic reports of the mammography were prospectively evaluated by one radiologist and reviewed (Selenia®, Hologic, Santiago, USA). The mammographic breast density was visually estimated according to the American College of Radiology Breast Imaging-Reporting and Data System classification and classified as follows: almost entirely fat (less than 25 % of breast comprising glandular tissue), having scattered fibroglandular densities (25–50 % of breast comprising glandular tissue), heterogeneously dense (51–75 % of breast comprising glandular tissue), and extremely dense (more than 75 % of breast comprising glandular tissue). Mammography positives were defined by an expert according to BI-RADS® Assessment Categories.

#### MRI review

Breast MRIs were performed on a 1.5 T system (Aera®, Siemens, München, Germany). Pre-contrast images of the dynamic series were subtracted from the post-contrast images to selectively highlight the enhancing structures. No parallel imaging was applied. MRI positives were defined by an expert as highly suspected and suggestive of biopsy or operation.

#### BSGI review

The patients were injected with 740–925 MBq (15–20 mCi) technetium-99 m sestamibi (Shanghai GMS Pharmaceutical Co., Ltd) into an arm vein. Craniocaudal and mediolateral views were performed of both breasts using a high-resolution, small field-of-view gamma camera optimized for breast imaging. Imaging was initiated immediately after injection of the isotope. Craniocaudal and mediolateral views were performed for both breasts with approximately 10 min per view (total time, 40 min). The images were obtained with a high-resolution, small field-of-view, breast-specific gamma camera (Dilon 6800 Gamma Camera; Dilon Technologies, Newport News, VA). BSGI positives were defined by an expert as highly suspected or having a tumor-to-normal tissue ratio (TNR) > 1.82.

### Data analysis

The statistical analyses were performed using SPSS, version 20. The comparison between BSGI and US, MMG and MRI, and BSGI diagnostic values for different clinicopathological variables were calculated using either χ^2^ tests with continuity correction or Fisher’s exact test. All statistical tests were two sided and considered significant when *p* ≤ 0.05.

## Results

### Patient characteristics

**Fig. 1:**
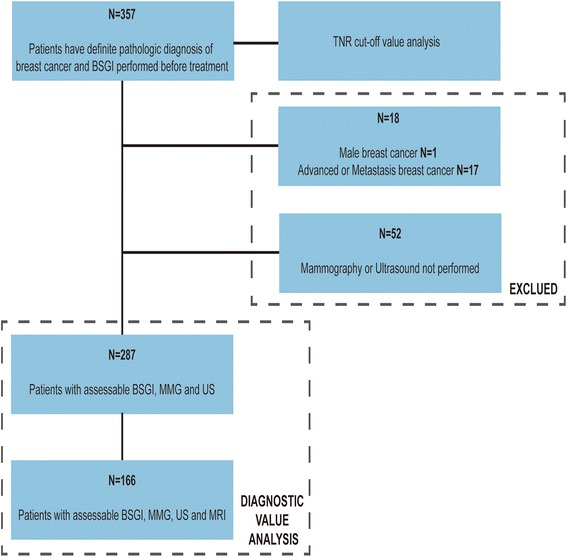
Flowchart of the study

There were 357 patients documented in our study. Of these, 287 patients underwent BSGI, US, and MMG, and 166 patients underwent all four imaging tests (BSGI, US, MMG and MRI) (Fig. 1). The median age of the study patients was 48.2 y and ranged from 32 to 75 y. The distribution of breast patient pathology was 168 malignant diseases (58.5 %), which was a combination of invasive ductal carcinoma (IDC), invasive lobular carcinoma (ILC), and ductal carcinoma in situ (DCIS), and 119 benign diseases (41.5 %).

### Cut-off values of TNR and distribution in breast malignant and benign disease

**Fig. 2:**
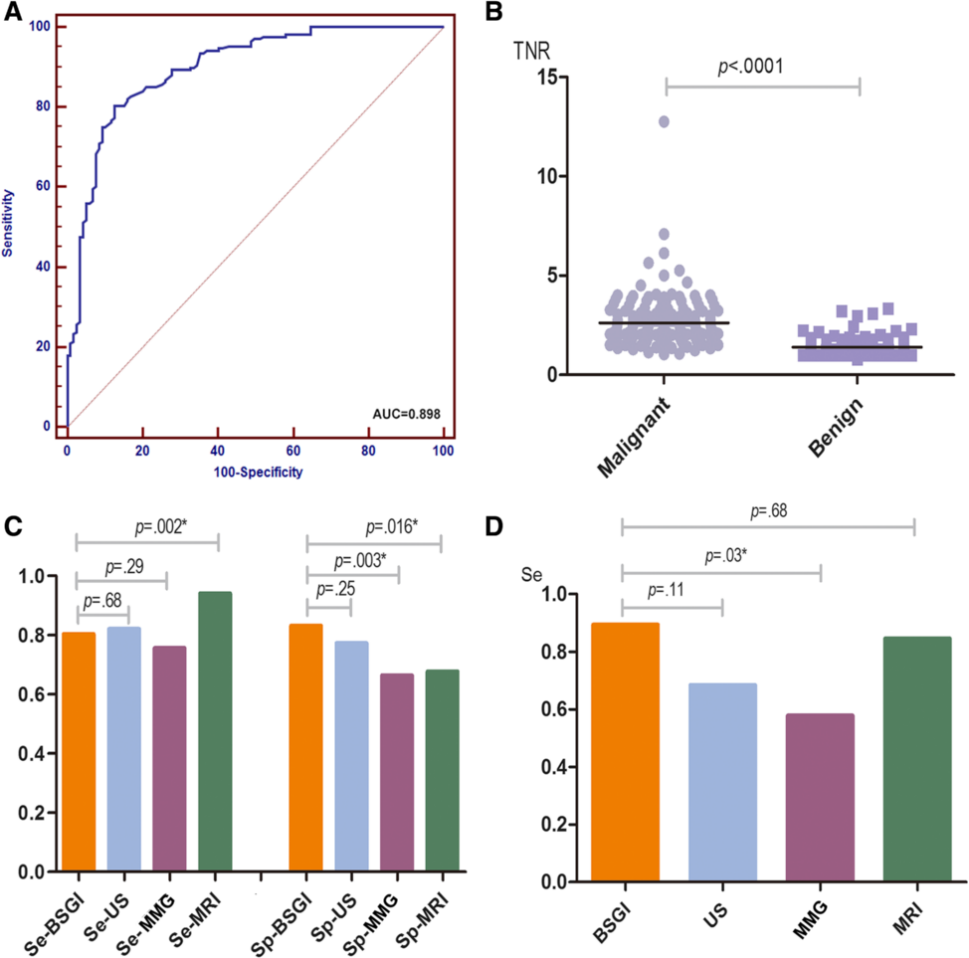
**a** ROC analysis for determining cut-off value of TNR in detection of breast cancer. **b** TNR of BSGI distribution in breast malignant and benign disease. **c** Sensitivity of BSGI, US, MMG and MRI for detecting breast cancer. **d** Sensitivity of BSGI, US, MMG and MRI for detecting DCIS

The cut-off value for TNR by sensitivity (Se), specificity (Sp) and Youden’s index (*YI*) analyses was 1.82 (Se:81.63 %, Sp:80.00 %, *YI*:61.63 %) (Fig. 2a). Our data also showed a statistically valid correlation for TNR between malignant breast diseases and benign diseases (*p* < 0.05). The mean TNR for the malignant group was 2.61 (95%CI 2.42–2.80), and for the benign group, the mean TNR was 1.41 (95 % CI 1.33–1.50) (Fig. 2b).

### Value of BSGI, US, MMG and MRI for detecting breast cancer

The sensitivity of MRI for detecting malignant lesions reached 94.06 % (95 % CI 87.01–97.56 %) and was superior to BSGI (80.35 %, 95 % CI 73.38–85.92 %), US (82.14 %, 95 % CI 75.33–87.45 %) and MMG (75.60 %, 95 % CI 68.26–81.74 %). However, the specificity of BSGI was the highest at 83.19 % (95 % CI 74.99–89.19 %) compared to US (77.31 %, 95 % CI 68.54–84.27 %), MMG (66.39 %, 95 % CI 57.07–74.63 %) and MRI (67.69 %, 95 % CI 54.82–78.46 %). Correspondingly, the positive-predictive value for the malignancy of a lesion, accuracy and Youden’s index (*YI*) were the highest for BSGI (87.10, 84.82 and 63.54 %, respectively). The *YI* for each imaging modality highlights the outstanding diagnostic potential of BSGI (63.54 %) compared to ultrasound (59.45 %), MMG (41.99 %) and MRI (61.75 %) for our diagnostic approach (Table 1 and Fig. 2c).

**Table 1:** Sensitivity and specificity of BSGI, US, MMG and MMRI for detecting breast cancer

	BSGI	US	MMG	MRI
	(%, 95 % CI)	(%, 95 % CI)	(%, 95 % CI)	(%, 95 % CI)
Se	80.35 % (73.38–85.92 %)	82.14 % (75.33–87.45 %)	75.60 % (68.26–81.74 %)	94.06 % (87.01–97.56 %)
Sp	83.19 % (74.99–89.19 %)	77.31 % (68.54–84.27 %)	66.39 % (57.07–74.63 %)	67.69 % (54.82–78.46 %)
PPV	87.10 % (80.54–91.75 %)	83.64 % (76.90–88.76 %)	76.05 % (68.72–82.15 %)	81.90 % (73.42–88.20 %)
NPV	75.00 % (66.57–81.94 %)	75.41 % (66.63–82.55 %)	65.83 % (56.55–74.09 %)	88.00 % (75.00–95.03 %)

There were 19 cases of DCIS. The sensitivity of BSGI for DCIS was 89.47 % (95 % CI 65.46–98.16 %) and 68.42 % for US (95 % CI 43.50–86.44 %), 57.89 % for MMG (95 % CI 33.97–78.88 %) and 84.62 % for MRI (95 % CI 53.66–97.29 %) (Fig. 2d).

When BSGI is combined with other examination techniques (MMG, US and MRI), we found that the accuracy for the detection of malignant breast lesions for BSGI combined with US was superior to BSGI + MMG or BSGI + MRI (Table 2).

**Table 2:** BSGI combined with other image techniques (MMG, US or MRI)

		US		MMG		MRI	
		Positive	Negative	Positive	Negative	Positive	Negative
BSGI	Positive	113	22	114	21	85	1
	Negative	25	8	13	20	10	5

The sensitivity and specificity for the detection of metastatic axillary lymph nodes by BSGI were 32 % (95 % CI 19.93–46.83 %) and 95.19 % (95 % CI 88.6–98.23 %), respectively (Table 3).

**Table 3:** BSGI for axillary lymph node staging in breast cancer

		Pathological Diagnosis		Se (%, 95 % CI)	Sp (%, 95 % CI)
		Positive	Negative		
BSGI	Positive	16	5	32	95.19
	Negative	34	99	(19.93–46.83)	(88.6–98.23)

### Sensitivity of BSGI, US, MMG and MRI in different traits of breast cancer

**Fig. 3:**
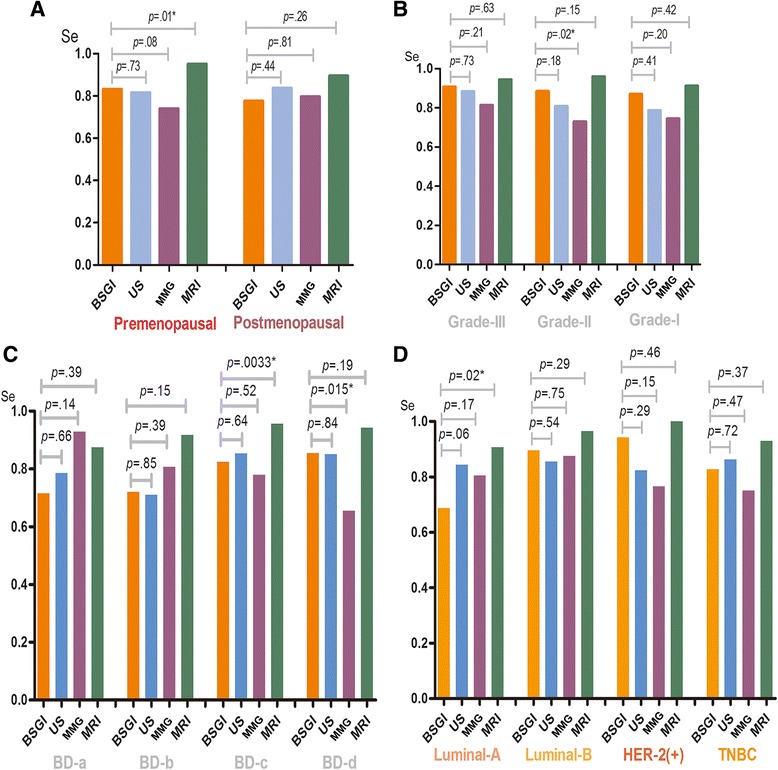
Sensitivity of BSGI, US, MMG and MRI in different traits of breast cancer

For premenopausal and postmenopausal women, the sensitivity of BSGI was not superior to breast US, MMG and MRI (Fig. 3a). The four breast density categories for breast composition are defined by the visually estimated content of fibroglandular dense tissue within the breasts. In the heterogeneously dense and extremely dense group, BSGI sensitivity was superior to MMG (82.35 % vs. 77.94 %; 85.45 % vs. 65.45 %, respectively) (Fig. 3c). For tumor grade and molecular subtype sensitivity analysis, the four imaging tests were not significantly different (Fig. 3b and d).

**Fig. 4:**
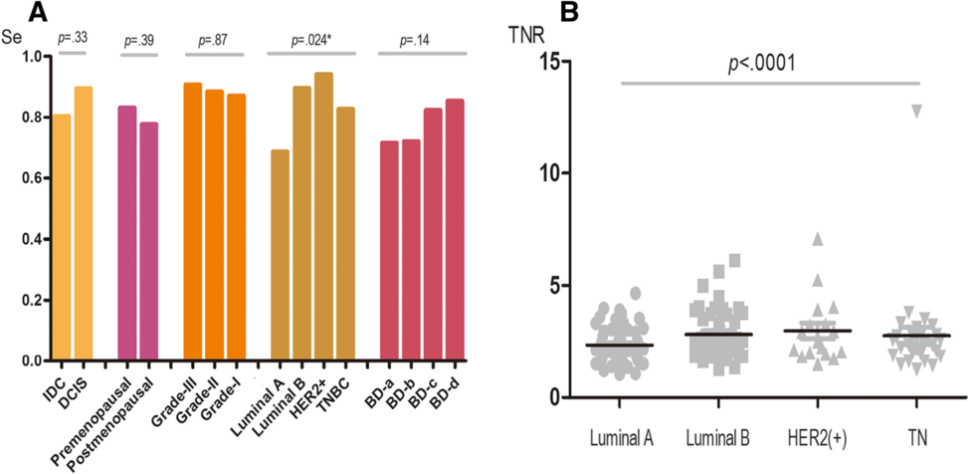
**a** Sensitivity of BSGI in different characteristics of breast cancer. **b** TNR of BSGI distribution for different breast cancer molecular subtypes

For the different breast cancer characteristics, the sensitivity of BSGI for detecting luminal A breast cancer was inferior at 68.63 % (95%CI 53.97–80.48 %) compared to luminal B (89.58 %, 95 % CI 76.56–96.10 %), HER-2(+) type (94.12 %, 95 % CI 69.24–99.69 %) and triple negative breast cancer (82.76 %, 95 % CI 63.51–93.47 %) (Fig. 4a). The TNR differed significantly between luminal-A and non-luminal-A breast cancer (*p* < .0001) (Fig. 4b).

### False-positive and false-negative findings of BSGI

We had 20 cases of false-positive BSGI findings. The pathology of false-positive BSGI lesions is shown in Table 4. We also analyzed the false-negative findings according to the breast cancer traits. The majority of false-negative malignant tumors had an extensive intraductal component (11/33), were high grade (9/33) and were diagnosed as Paget’s disease (2/33).

**Table 4:** Analysis of BSGI false positive in diagnosis of breast cancer

	Number	Percent
Intraductal Papilloma	7	35
Sclerosing Adenosis	3	15
Fibroadenoma	3	15
Fibrocystic Disease	2	10
Breast Cyst	2	10
Chronic Inflammation	2	10
Benign Phylloides Tumor	1	5

## Discussion

To our knowledge, this study is the first to evaluate the diagnostic value of breast specific gamma imaging for Chinese women. We found that BSGI could be used in a work-up of suspicious breast lesions. The visual and semi-quantitative analyses (TNR cut-off value 1.82) as combined for detecting primary breast cancer [15]. Comparison of the sensitivity and specificity of BSGI to US, MMG and MRI for breast cancer diagnosis, the values for BSGI were 80.35 and 83.19 %, respectively, and were slightly higher than for the other imaging tests (US, MMG and MRI). For Chinese breast cancer patients, approximately 73.72 % (123/168) of patients have heterogeneously dense or extremely dense breasts, and BSGI had significantly higher sensitivity compared to MMG, indicating that BSGI is rarely affected by breast density. For different molecular subtypes, non-luminal A types have a higher degree of sensitivity by BSGI examination.

### Diagnostic value of BSGI

For breast cancer detection sensitivity and specificity, BSGI is superior to US and MMG, and showed equal sensitivity and high specificity compared to MRI for the diagnosis of breast lesions. According to tumor type, BSGI had good sensitivity for discriminating DCIS (89.47 %), suggesting that BSGI may play a crucial role as an adjunctive imaging modality to evaluate breast ductal carcinoma in situ. For Chinese women, almost 75 % have dense breasts, limiting MMG screening (sensitivity 75.60 %, specificity 66.39 %). BSGI was not influenced by breast density, and the sensitivity of heterogeneously dense or extremely dense breast patients was 82.35 and 85.45 %, respectively. Recently, studies indicated that the sensitivity of BSGI for detecting subcentimeter (<1 cm) breast cancer was 84 % (95 % CI 80–88 %) [16, 17]. Combined with our research, evidence suggests that BSGI, as a functional imaging test, is an extremely useful adjunct test for its ability to identify breast cancer with high diagnostic performance, and it was not influenced by menstrual state, tumor grade, or tumor size [16, 18, 19]. Due to the limitations of the examination methods, axilla are hard to test, but BSGI has a higher specificity for detecting axillary lymph node metastasis [20].

Screening mammography has been the gold standard for breast cancer detection for the past 30 years [21, 22], but recent studies have questioned this screening because it does not reduce breast cancer mortality [23]. MRI is currently recommended by the American Cancer Society in patients with high risk, but there are issues with sensitivity resulting in increased false positive rates leading to numerous benign biopsies or operations [24]. Studies demonstrated that BSGI has an equal sensitivity with a higher specificity than MRI as an adjunct imaging modality for the diagnosis of breast cancer. Additional advantages include greater ease of imaging for the patient, lower cost, and an easy read for the radiologist or surgeon [18, 25, 26]. Specifically in China, women have dense mammary glands and BSGI will show a higher value in the current paradigm of breast imaging for screening and diagnosis. First, for breast patients with BI-RADS® 0 or 3 by US and/or MMG, high risk, and/or MMG dense breasts, BSGI was a useful adjunctive imaging method to reduce the false-negative rate (missed diagnosis rate). Second, for breast patients with BI-RADS® 4 by US and/or MMG, biopsy is recommended. BSGI can reliably identify the US and/or MMG findings that are benign, which can avoid unnecessary biopsies for a majority of patients. Therefore, BSGI is highly recommended in these two situations for Chinese women.

### Characterization of breast lesions with BSGI

**Fig. 5:**
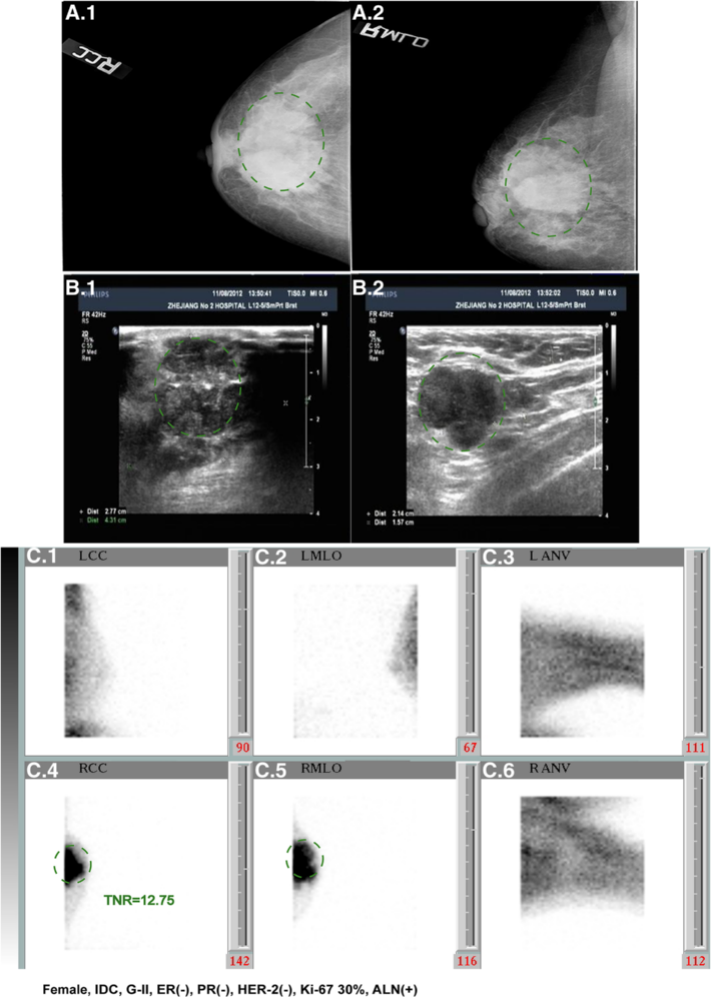
Images for the case of breast cancer patient with TRN = 12.75. **a** MMG for right breast. **b** US for right breast and axillary. **c** BSGI image

As a functional imaging, semi-quantitative analysis is an important parameter of BSGI, which reflects the Tc-99 m MIBI uptake level. We found malignant lesions have a higher TNR than benign lesions (mean 2.61 vs. 1.41, *p* < .0001). This makes the semi-quantitative value of BSGI in breast cancer diagnosis possible. Interestingly, one case had a particularly high TNR, with TNR = 12.75, and we reviewed this patient’s medical files. This was a 50-year-old menopausal female, and the imaging examination is shown in Fig. 5. The pathologic diagnosis was invasive ductal carcinoma, WHO II grade. IHC tests showed that the tumor was ER negative, PR negative, HER-2 negative, and 30 % Ki-67 positive. After biopsy, this patient received standardized therapy and follow-up. At 20 months after diagnosis, this patient died because of the rapid spread of cancer with pulmonary metastasis and malignant pleural effusion. This case suggests that a high value of TNR may correlate with a poor prognosis [27].

For different molecular subtypes, the data show that the luminal A type has a lower TNR value (mean 2.35, 95 % CI 2.11–2.59) compared to the luminal B type (mean 2.82, 95 % CI 2.51–3.14), HER-2 positive type (mean 2.99, 95 % CI 2.24–3.73) and triple negative type (mean 2.77, 95 % CI 2.00–3.54). For BSGI diagnosis, the sensitivity of the luminal A type was the lowest (68.63 % 95 % CI 53.97–80.48 %), whereas the HER-2 positive type had the highest sensitivity (94.12 % 95 % CI 69.24–99.69 %) compared to the other subtypes (luminal B: 89.56 % 95 % CI 76.56–96.10 %; TNBC: 82.76 % 95 % CI 63.51–93.47 %). Therefore, BSGI may help classify the sub-type of an invasive ductal carcinoma in addition to its pathology.

### Limitations of BSGI

BSGI has several limitations as a breast imaging modality. Patients are exposed to radiation from the BSGI test of approximately 6.29–9.44 mSv [8, 28]. BSGI may be recommended for patients with suspicious breast lesions or dense breasts by conventional methods, using lower doses and longer acquisition times [29]. Second, this is a plane test, and there may be insufficient positioning [16, 30]. Finally, BSGI was the least sensitive for detecting axillary lymph nodes.

## Conclusion

In summary, BSGI showed a borderline sensitivity but a higher specificity than US/MMG/MRI for diagnosing breast lesions, and it has a high sensitivity for discriminating DCIS. BSGI may play a crucial role in discriminating breast lesions and can be used to evaluate newly diagnosed breast cancer patients with dense breasts. Semi-quantitative analysis as a parameter of BSGI may help classify the sub-type of an invasive ductal carcinoma in addition to the pathology. Because Chinese women have unique breast density, BSGI may improve the early detection of breast cancer to reduce breast cancer morbidity and mortality.

## Abbreviations

BSGI, breast specific gamma imaging; MMG, mammography; MRI, magnetic resonance imaging; Se, sensitivity; Sp, specificity; US, ultrasound; YI, yueden’s index
